# Diversity of Shiga toxin transducing phages in *Escherichia coli* O145:H28 and the different Shiga toxin 2 production levels associated with short- or long-tailed phages

**DOI:** 10.3389/fmicb.2024.1453887

**Published:** 2024-08-06

**Authors:** Keiji Nakamura, Itsuki Taniguchi, Yasuhiro Gotoh, Junko Isobe, Keiko Kimata, Yukiko Igawa, Tomoko Kitahashi, Yohei Takahashi, Ryohei Nomoto, Kaori Iwabuchi, Yo Morimoto, Sunao Iyoda, Tetsuya Hayashi

**Affiliations:** ^1^Department of Bacteriology, Graduate School of Medical Sciences, Kyushu University, Fukuoka, Japan; ^2^Toyama Institute of Health, Imizu, Japan; ^3^Nagano Prefecture Suwa Public Health and Welfare Office, Suwa, Japan; ^4^Chiba City Institute of Health and Environment, Chiba, Japan; ^5^Aomori Prefectural Institute of Health, Aomori, Japan; ^6^Kobe Institute of Health, Kobe, Japan; ^7^Iwate Prefectural Research Institute for Environmental Sciences and Public Health, Morioka, Japan; ^8^Hokkaido Institute of Public Health, Sapporo, Japan; ^9^National Institute of Infectious Diseases, Tokyo, Japan

**Keywords:** Shiga toxin-producing *Escherichia coli*, Stx phage, Stx2 production, O145:H28, comparative genomics

## Abstract

Shiga toxin (Stx)-producing *Escherichia coli* (STEC) causes serious gastrointestinal illness, including hemorrhagic colitis and hemolytic uremic syndrome. Two types of Stxs (Stx1 and Stx2) are known and both are encoded by bacteriophages (Stx phages), but the production of Stx2 is known to be a major risk factor for severe STEC infections. The production of Stx2, but not Stx1, is tightly coupled with the induction of Stx phages, and Stx2 production levels vary between STEC strains even within the same serotype. Here, we analyzed the genomic diversity of all Stx phages in 71 strains representing the entire O145:H28 lineage, one of the often highly pathogenic STECs, and the relationship between the variations in Stx phage genomes and the levels of Stx2 production by host strains. Our analysis reveals highly dynamic natures of Stx phages in O145:H28, including the independent acquisition of similar Stx phages by different sublineages, the recent transfer of Stx phage between different sublineages, and the frequent gain and loss of Stx phages in some sublineages. We also show the association of the Stx2 phage types with the Stx2 production levels of host strains: strains carrying short-tailed Stx2 phages exhibited significantly higher Stx2 production levels than those carrying long-tailed Stx2 phages. Detailed analyses of the Stx2 phage genomes revealed that both of short- and long-tailed phages exhibited sequence diversification and they were divided into two groups, respectively, based on the sequence similarity of the phage early region encoding genes responsible for phage induction, short-tailed phages contained early regions clearly different in genetic organization from those in long-tailed phages. Therefore, the variations in the early regions between short-and long-tailed Stx2 phages appeared to be linked to a striking difference in Stx2 production levels in their host strains. These results broaden our understanding of the diversification and dynamism of Stx phages in O145:H28 and the association of Stx2 phage types with the Stx2 production level in this STEC lineage.

## Introduction

Shiga toxins (Stxs) are the key virulence factors of Stx-producing *Escherichia coli* (STEC), which causes diarrhea and hemorrhagic colitis with life-threatening complications, such as hemolytic uremic syndrome. Stxs are classified as Stx1 or Stx2, each of which include several subtypes (Stx1a, Stx1c-Stx1e; Stx2a-Stx2l) (Scheutz et al., 2012; Koutsoumanis et al., 2020). While STEC strains produce one or more Stx subtypes (Ogura et al., 2007; Steyert et al., 2012), epidemiological studies suggest that Stx2-producing strains cause more severe STEC infections than strains producing only Stx1 (Fuller et al., 2011; Bielaszewska et al., 2013).

The *stx* genes are encoded by bacteriophages (Stx phages), and STEC strains acquire these genes via the lysogenization of Stx phages. Although the integration sites and genome sequences of Stx phages are highly variable even within the same serotype (Delannoy et al., 2017; Yara et al., 2020; Miyata et al., 2023; Yano et al., 2023), Stx phages are morphologically divided into two types based on their tail structures, which are defined by late genes: lambda-like long-tailed phages (L-phages) and short-tailed phages (S-phages) similar to the Stx2a phages of O157:H7, such as Sp5 and 933W (Plunkett et al., 1999; Teel et al., 2002; Muniesa et al., 2004; Strauch et al., 2004; Asadulghani et al., 2009; García-Aljaro et al., 2009; Bonanno et al., 2016; Pinto et al., 2021). Of 279 publicly available Stx phages, 99% can be classified into either type based on genetic content (Pinto et al., 2021). Both types of Stx phages encode several lambda-like regulator genes that modulate early and late gene expression, such as the *cI* and *q* genes (Neely and Friedman, 1998; Makino et al., 1999; Plunkett et al., 1999; Strauch et al., 2008).

The *stx* genes are located downstream of the late gene promoter (Plunkett et al., 1999; Yokoyama et al., 2000; Shi and Friedman, 2001; Strauch et al., 2008). The expression of *stx1* is primarily under the control of the iron-regulated authentic promoter (Calderwood and Mekalanos, 1987), although prophage induction-dependent production of Stx1 has been described in some O157:H7, O26:H11, and O48:H21 strains (Wagner et al., 2002; Steyert et al., 2012; Berger et al., 2019; Yano et al., 2023). In contrast, the expression of *stx2* depends on the late promoter (Wagner et al., 2001; Tyler et al., 2004) and Stx2 production is tightly coupled with phage induction; thus, variations in Stx2 phage genomes can affect the amount of Stx2 production by each strain, as has been shown in several STEC lineages (Ogura et al., 2015; Nishida et al., 2021; Miyata et al., 2023; Nakamura et al., 2023). For example, the variation in Stx2 production levels in O157:H7 STEC strains was associated with the subtypes of Stx2a phages (all are S-phages) as defined by their early regions (Ogura et al., 2015). In particular, in O157:H7 clade 8, a highly pathogenic lineage of O157:H7 STEC, the γ subtype of the Stx2a phage confers increased Stx2 production and pathogenicity to host strains than do other clade 8 strains, which carry the Stx2a phages belonging to the δ subtype (Miyata et al., 2023).

O145:H28 is one of the often highly pathogenic STECs (Karmali et al., 2003; Valilis et al., 2018). We previously analyzed the whole-genome sequences (WGSs) of 239 O145:H28 strains, including a systematic analysis of the prophages in seven finished genomes, and revealed notable variations in the sequences and integration sites of Stx phages among O145:H28 strains (Nakamura et al., 2020). In that study, we found that although the distribution of *stx1a* genes was biased toward specific clades, the *stx2a* genes were widely but variably distributed throughout the entire O145:H28 lineage. However, the precise variation in Stx phages and its impact on Stx production by host strains of this lineage have not been elucidated. In the present study, we performed a systematic analysis of Stx phages of STEC O145:H28 and analyzed the diversity and dynamism of Stx phages in O145:H28 and the association of Stx2 phage types with Stx2 production by host strains.

## Materials and methods

### Bacterial strains

The initial O145:H28 strain set included 59 strains that were available in our laboratory and sequenced in our previous study (Nakamura et al., 2020) and 18 strains with complete genome sequences (the plasmid genome was not available for strain 2015C-3125). The genome sequences of the 18 previously reported strains (Cooper et al., 2014; Patel et al., 2018; Tyson et al., 2019; Carter et al., 2021) were downloaded from NCBI. Six of the 18 strains were excluded from the strain set because their recombination-free core genome sequences were identical to those of other strains. Therefore, in this study, 71 strains were analyzed (see Supplementary Table S1 for the strains included in the final set).

### Determination of complete genome sequences

The genomic DNA of strains 16003 and 12E115 was purified using Genomic-tip 100/G (Qiagen). Libraries for Illumina sequencing (average insert size: 700 bp) were prepared using the NEBNext Ultra II FS DNA Library Prep Kit (New England Biolabs) and sequenced using Illumina MiSeq to generate 300 bp paired-end reads. These genomes were additionally sequenced using MinION with R9.4.1 flow cells (Nanopore) for 68 (16003) or 96 h (12E115). MinION reads were trimmed and filtered as described previously (Nishida et al., 2021) and assembled along with the Illumina reads of each strain using Unicycler v0.4.8 (Wick et al., 2017) to obtain the finished genome sequences.

### Phylogenetic analysis

Phylogenetic analysis of the initial strain set (*n* = 77) was performed based on the SNPs identified on the prophage/integrative element/IS-free and recombination-free chromosome backbone that was conserved across all genomes by Gubbins (Croucher et al., 2015) and MUMmer (Kurtz et al., 2004) using the genome of strain 10942 as a reference. A maximum likelihood (ML) tree was constructed with RAxML v8.2.12 (Stamatakis, 2006) as previously described (Nakamura et al., 2021) and displayed using FigTree v1.4.4.^1^

### Analyses of integration sites and the sequencing of Stx phages

Stx phages integrated into the *attB* in the *ompW* prophage and *yecE* loci in 54 O145:H28 draft genomes were previously described (Nakamura et al., 2021). Stx phages integrated into *argW*, *wrbA*, and *sbcB* in these 54 genomes were identified by the same strategy as that we previously employed. Briefly, we first examined each site by BLASTN search, and when some phage (or other genetic element) was found to be integrated into the site, long PCR amplification using primers targeting the *stx* genes and sequences adjacent to the integration site was performed to determine whether the integrated phage was Stx phage or not (schematically shown in Supplementary Figure S1; see Supplementary Table S2 for the primers used). The entire genome sequence of each phage was determined by sequencing long PCR products as previously described (Nakamura et al., 2021). DFAST v1.2.18 (Tanizawa et al., 2018) and GenomeMatcher v3.0.2 (Ohtsubo et al., 2008) were used for annotating phage genomes and comparing Stx phage genomes, respectively. Based on the late gene organization, Stx phages that contained late genes similar to those of phage lambda were defined as L-phages and Stx phages that contained late genes similar to those of Sp5 and 933W were defined as S-phages.

### Clustering analyses of Stx phage genomes and sequence analysis of Q proteins

All-to-all phage genome comparison was performed for the three sets of genomes (the entire genomes of 83 Stx phages, the entire genomes of 54 Stx2 phages, and the early regions of 54 Stx2 phages) using Mash v2.0 (Ondov et al., 2016) with default parameters to generate pairwise Mash distance matrices. Based on each matrix, Stx phages and the early regions of Stx phages were clustered with a cutoff of 0.05 as previously described (Nagano et al., 2023). The amino acid sequences of Q proteins encoded by each Stx2 phage were aligned with the Q proteins from phages lambda, Sp5, and 933W by MUSCLE in MEGA v10.1.8 (Kumar et al., 2018), and a dendrogram was generated based on the alignment using the UPGMA algorithm in MEGA v10.1.8.

### Determination of Stx2 production levels

Cell lysates of all tested strains were prepared as described previously (Nishida et al., 2021), except for the final concentration (1.0 μg/mL) of mitomycin C (MMC; Wako Chemicals). The MMC concentration and sampling time were optimized based on the results of exploratory analyses using seven O145:H28 strains (see Supplementary Figure S2 for details). The Stx2 concentration in the lysate of each O145:H28 strain was determined by sandwich ELISA as previously described (Nishida et al., 2021). An unpaired *t* test was performed to compare Stx2 production levels between the O145:H28 strains carrying S-Stx2a phages and those carrying L-Stx2 phages using Prism 9 software (GraphPad Software). *p* < 0.05 was considered to indicate statistical significance.

## Results and discussion

### Strain set and Stx phages

**Figure 1:**
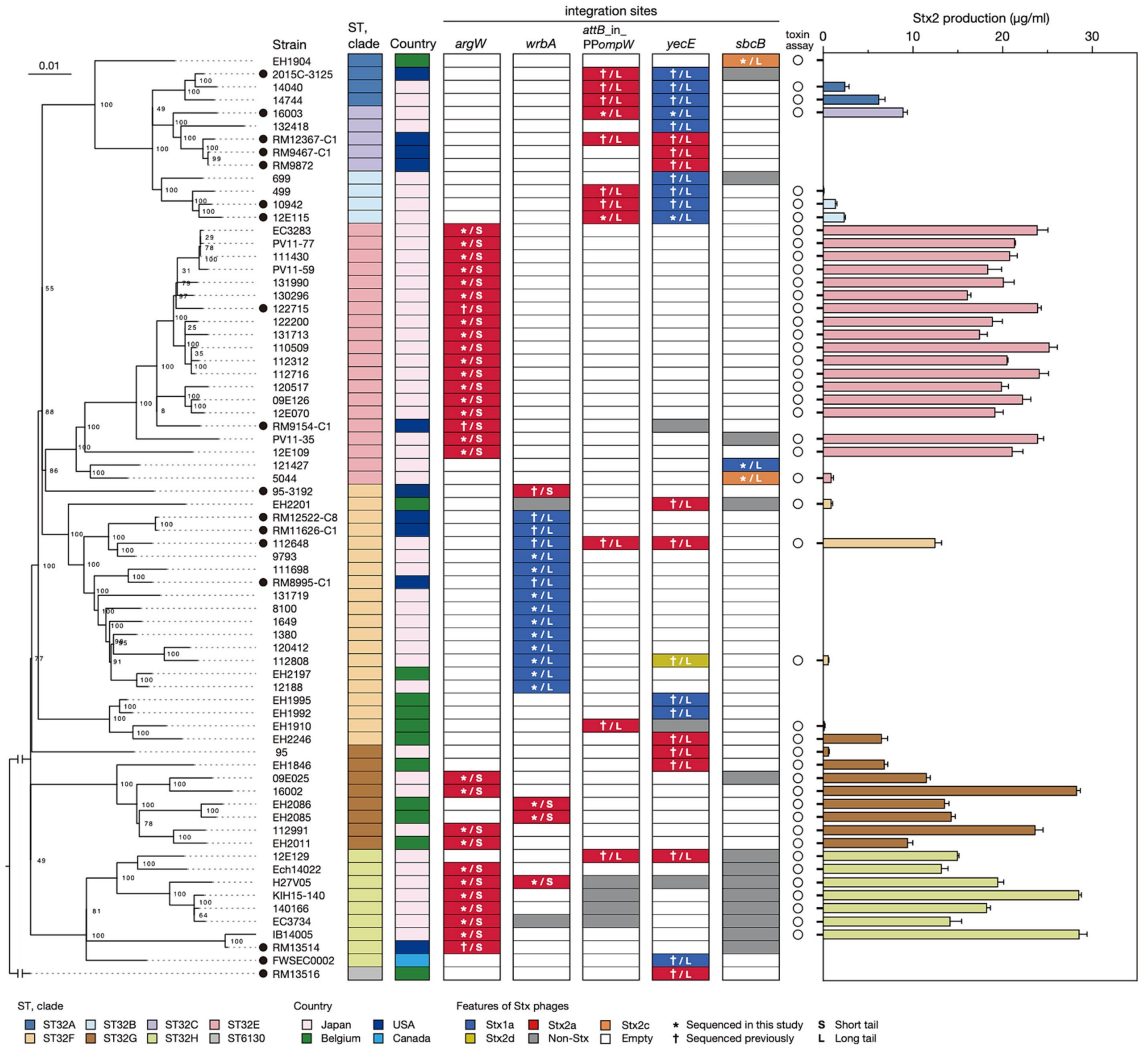
Variation in the integration site of Stx phages and the Stx2 production level in STEC O145:H28 strains. The phylogenetic tree of 71 O145:H28 strains is shown in the left panel. The tree was constructed based on the recombination-free SNPs (3,347 sites) that were identified on the conserved chromosome backbone (3,851,013 bp) by RAxML using the GTR gamma substitution model. The reliabilities of the tree’s internal branches were assessed by bootstrapping with 1,000 pseudoreplicates. The bar in the upper-left corner indicates the mean number of nucleotide substitutions per site. Genome-finished strains are indicated by filled circles. Along with the tree, the geographic and ST/clade information of strains, the presence or absence of prophages at five loci, and the features of prophages are shown. In the right panel, the levels of MMC-induced Stx2 production by each strain are shown as the mean values with standard errors of biological triplicates. Note that the Stx2 production levels of eight *stx2*-positive strains whose genome sequences were obtained from NCBI were not determined.

For detailed analyses of Stx phages in O145:H28 strains, we selected 64 strains from the 239 strains analyzed in our previous study (Nakamura et al., 2020), 59 of which were sequenced in our laboratory. This set included eight finished and 56 draft genomes and covered seven of the eight clades in ST32 (clades A-H) and the ST137/ST6130 lineage in O145:H28 (ST32 clade D strains were not available in our laboratory). Among the 56 strains, for which only draft genomes were available, two were subjected to Nanopore long-read sequencing to obtain finished sequences by hybrid assembly. Seven genome-finished strains recently deposited in the NCBI database (Tyson et al., 2019; Carter et al., 2021) were also included in the dataset (Supplementary Table S1). Thus, the final set included 71 strains (Figure 1 and Table 1), of which 48 were isolated in Japan and the remainder were isolated in the USA, Belgium, or Canada. Most strains were human isolates, but five bovine and four environmental/food isolates were included. There were five *stx* genotypes, and four strains carried two copies of the *stx2a* gene (Table 1).

**Table 1:** The *stx* genotypes of the O145:H28 strain analyzed in this study.

Country	*stx1a*	*stx1a stx2a*	*stx1a stx2a* (×2)^a^	*stx1a stx2d*	*stx2a*	*stx2a* (×2)^a^	*stx2c*	Total
Japan	11	6	1	1	26	2	1	48
United States	3	1	0	0	5	1	0	10
Belgium	3	0	0	0	8	0	1	12
Canada	1	0	0	0	0	0	0	1
Total	18	7	1	1	39	3	2	71

a 2 copies of *stx2a* genes.

As the genome sequences of the Stx phages of 27 strains were already known (*n* = 38), we determined those of the Stx phages (*n* = 45) in the remaining 43 strains. The Stx1a phage in an *stx1a*/*stx2d*-positive strain (strain 112808, whose Stx2d phage was previously sequenced but Stx1a phage was not) was also sequenced to obtain the full set of genome sequences of Stx phages (*n* = 84) and determine their integration sites (Figure 1 and Supplementary Table S1).

Of the 84 phages, 33 were S-Stx2a phages, and 21 were L-Stx2a phages. The remaining 30 phages encoded other types of Stxs, and all were L-phages (27 Stx1a, two Stx2c, and one Stx2d). For the integration sites, five loci (*argW*, *wrbA*, *attB* in the *ompW* prophage, *yecE*, and *sbcB*) were identified. The L-Stx2a phage was duplicated in strain 112648 and integrated into the *attB* site in the *ompW* prophage (referred to as *attB*_in_PP*ompW*) and the *yecE* loci (Nakamura et al., 2021); thus, these phages were considered one L-Stx2a phage. Although three strains (12E129, RM12367-C1, and H27V05) contained two Stx2a phages, they were included as different phages because they showed considerable sequence variation (Supplementary Figure S3); thus, a total of 83 Stx phages were analyzed in subsequent analyses.

### Dynamics of Stx phages in O145:H28 isolates

**Figure 2:**
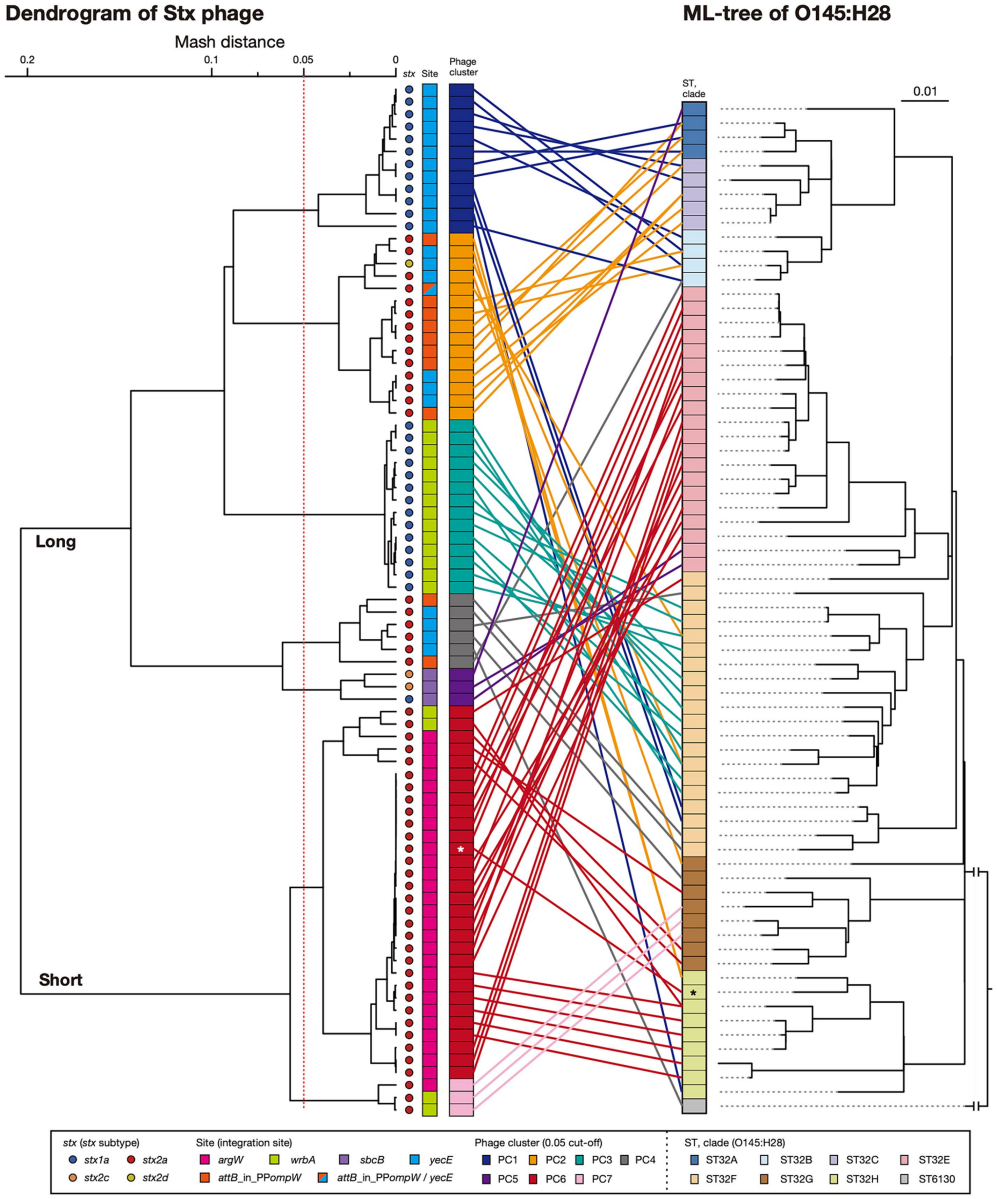
Sequence similarities among Stx phages found in the 71 O145:H28 strains. A dendrogram based on the Mash distance matrix of 83 Stx phage genomes is shown in the left panel, along with their *stx* genotypes, integration sites, and phage clusters, which were defined based on the pairwise Mash distance with a cutoff distance of 0.05. The phage indicated by *attB*_in_PP*ompW*/*yecE* was the duplicated L-Stx2a phages that were integrated into the *attB*_in_PP*ompW* and *yecE* loci in strain 112648. These duplicated phages were treated as one phage. The tree in the right panel is the same ML tree of O145:H28 strains shown in Figure 1. Stx phages were connected to their host strains by lines colored according to the phage clusters. Strain Ech14022 and its S-Stx2a phage are indicated by asterisks.

To analyze the sequence similarities of the 83 Stx phages, we performed all-to-all sequence comparisons using the Mash program (Ondov et al., 2016) and constructed a dendrogram using the complete linkage method based on pairwise Mash distances. The 83 phages were clearly divided into L-phages and S-phages (Figure 2 and Supplementary Table S3), but multiple phage clusters were detected with a threshold of 0.05 in both types of phages (PC1-PC5 in L-phages and PC6 and PC7 in S-phages).

In five phage clusters (PC1, 3, 4, 6, and 7), the encoded *stx* was the same subtype. However, variations in *stx* subtype were found in PC2 and PC5 (12 *stx2a* and one *stx2d* in PC2 and one *stx1a* and two *stx2c* in PC5), suggesting the replacement of *stx* in these two phage clusters. While the Stx1a phages at *yecE* and *wrbA* (PC1 and PC3) and the Stx1a and Stx2c phages at *sbcB* (PC5) formed distinct clusters, the L-Stx2a and S-Stx2a phages were separated into two clusters, respectively (Figure 2). Interestingly, although the L-Stx2a phages were separated into PC2 and PC4, both included phages at *attB*_in_PP*ompW* and *yecE*. Similarly, S-Stx2a phages were separated into PC6 and PC7, but both included phages at *argW* and *wrbA*. Variations in PC2 and PC4 can be easily generated because the *attB* sequences in *attB*_in_PP*ompW* and *yecE* are essentially the same (Nakamura et al., 2021). In contrast, the variation in PC2 and PC4 was apparently generated by replacement of the integrase gene.

The within-cluster heterogeneity of Stx phages was more evident when the phylogeny of their host strains was considered (Figure 2). While two clusters (PC3 and PC7) were comprised of phages found in the same host clade, respectively, the remaining five clusters included Stx phages found in multiple host clades. For example, PC1 phages were found in strains belonging to clades A, B, C, F, and H, and PC2 phages were found in clades A, B, C, F, G, and H. This finding indicates dynamic changes in Stx phages in each clade. The most striking case was the S-Stx2a phage of strain Ech14022 of clade H (indicated by an asterisk in Figure 2), which had a sequence nearly identical to those of the S-Stx2a phages of clade E strains, suggesting the recent interclade transfer of this phage.

These findings suggest that wide circulation of each phage cluster likely allowed the acquisition of similar phages by host strains with various phylogenetic backgrounds. Repeated acquisition of Stx phages may induce the loss of resident Stx phages, leading to within-host clade variation in Stx phages. Similar changes/variations were observed for Stx1a phages in the ST21 lineage of STEC O26:H11 (Yano et al., 2023) and Stx2a phages in clade 8 of STEC O157:H7 (Miyata et al., 2023). Notably, however, different distribution patterns of Stx phages were also observed in STEC O121:H19 and the STEC belonging to clonal complex 119 (CC119). In these STECs, systematic analyses of Stx phages have been conducted and revealed the stable maintenance of an S-Stx2a phage and an L-Stx2a phage in the major lineage of each STEC, respectively (Nishida et al., 2021; Nakamura et al., 2023).

### Stx2 production was greater in the strains carrying S-Stx2 phages than in the strains carrying L-Stx2 phages

**Figure 3:**
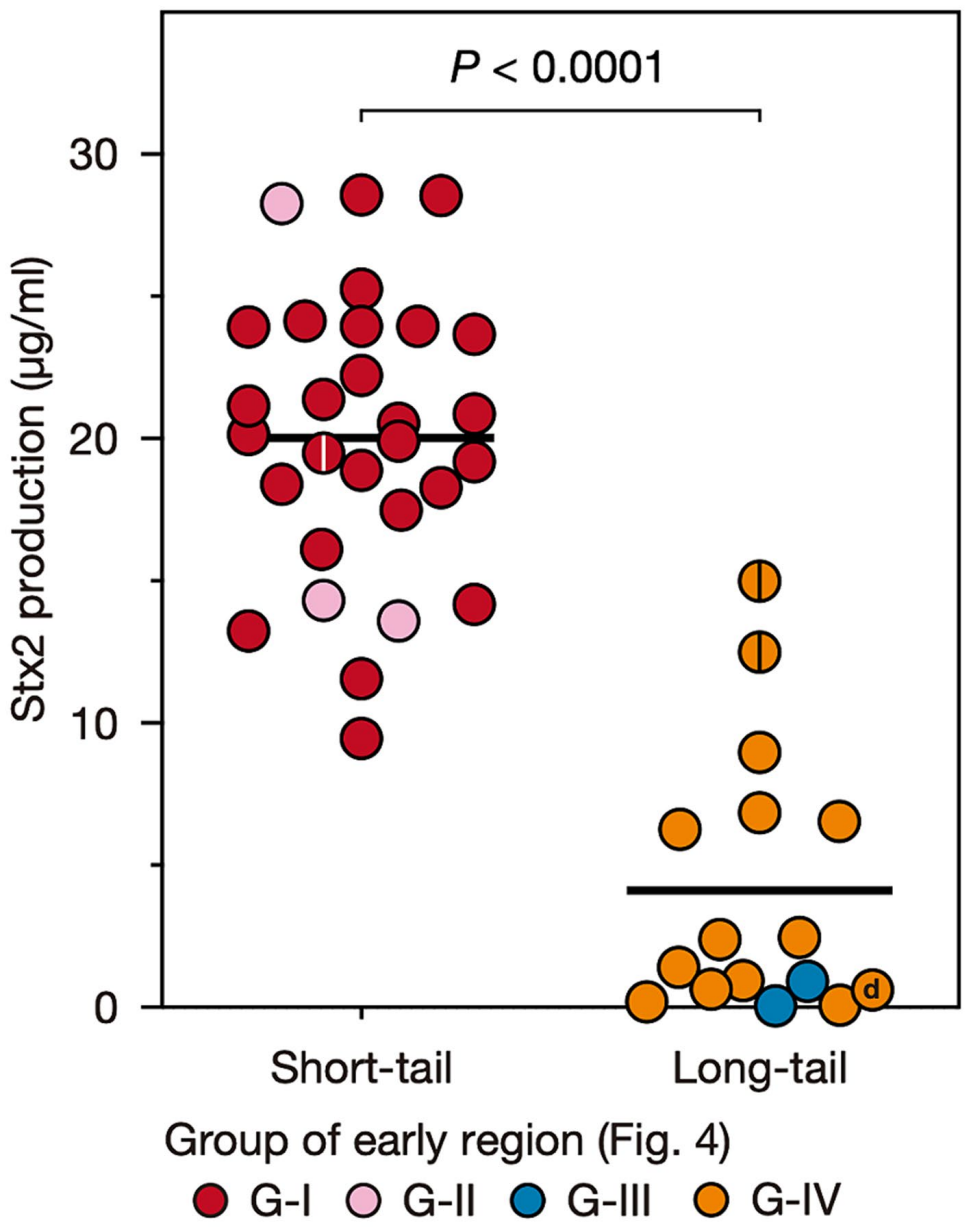
Stx2 production levels of O145:H28 strains harboring S-Stx2a phages and those harboring L-phages encoding Stx2a, Stx2c, or Stx2d. Most L-phages encoded Stx2a (L-Stx2a phages), but two encoded Stx2c and one encoded Stx2d. The Stx2 production level of each strain was presented as the mean value of biological triplicates. Each strain is colored according to the groups defined based on the sequence similarity of the early region of their Stx2 phages shown in Figure 4. Stains carrying two Stx2a phages are indicated by center lines in circles. The Stx2d phage-carrying strain is indicated by “d”.

To examine the variation in the level of MMC-induced Stx2 production across O145:H28 strains, we optimized the MMC concentration and sampling time using seven O145:H28 strains (Supplementary Figure S2) and measured the Stx2 concentrations using this condition in the cell lysates of 45 *stx2*-positive strains available in our laboratory (29, 13, 2, and 1 strains carried S-Stx2a, L-Stx2a, L-Stx2c, and L-Stx2d phages, respectively), which covered seven of the eight clades in ST32. As shown in Figure 1 and Supplementary Table S3, the Stx2 production levels were highly variable between the strains (0.06–28.6 μg/mL). Moreover, the comparison of strains carrying S-Stx2a phages and those carrying L-Stx2 phages (including Stx2a, Stx2c, and Stx2d phages) revealed that the former strains produced significantly more Stx2 than the latter strains (19.8 vs. 4.1 μg/mL on average, *p* < 0.0001) (Figure 3). The Stx2 production level of strain H27V05, which carried two S-Stx2a phages, was average (19.5 μg/mL) among the S-Stx2a phage-carrying strains. However, the two strains (112648 and 12E129) that carried two L-Stx2a phages produced greater amounts of Stx2 (12.6 μg/mL and 15.0 μg/mL, respectively) than the other strains carrying L-Stx2 phages. It has recently been reported that two O113:H21 strains carrying L-Stx2 phages showed a significant difference in Stx2 production level and the strain producing a greater amount of Stx2 carried two L-Stx2 phages (Allué-Guardia et al., 2022). Although this finding may be linked to our finding, it should be noted that these two O113:H21 strains belonged to different *E. coli* lineages and the two strain L-Stx2 phages of the strain showing a higher Stx2 production level encoded the *stx2a* and *stx2d* genes, respectively.

### Variations in the early genes of S- and L-Stx2 phages

**Figure 4:**
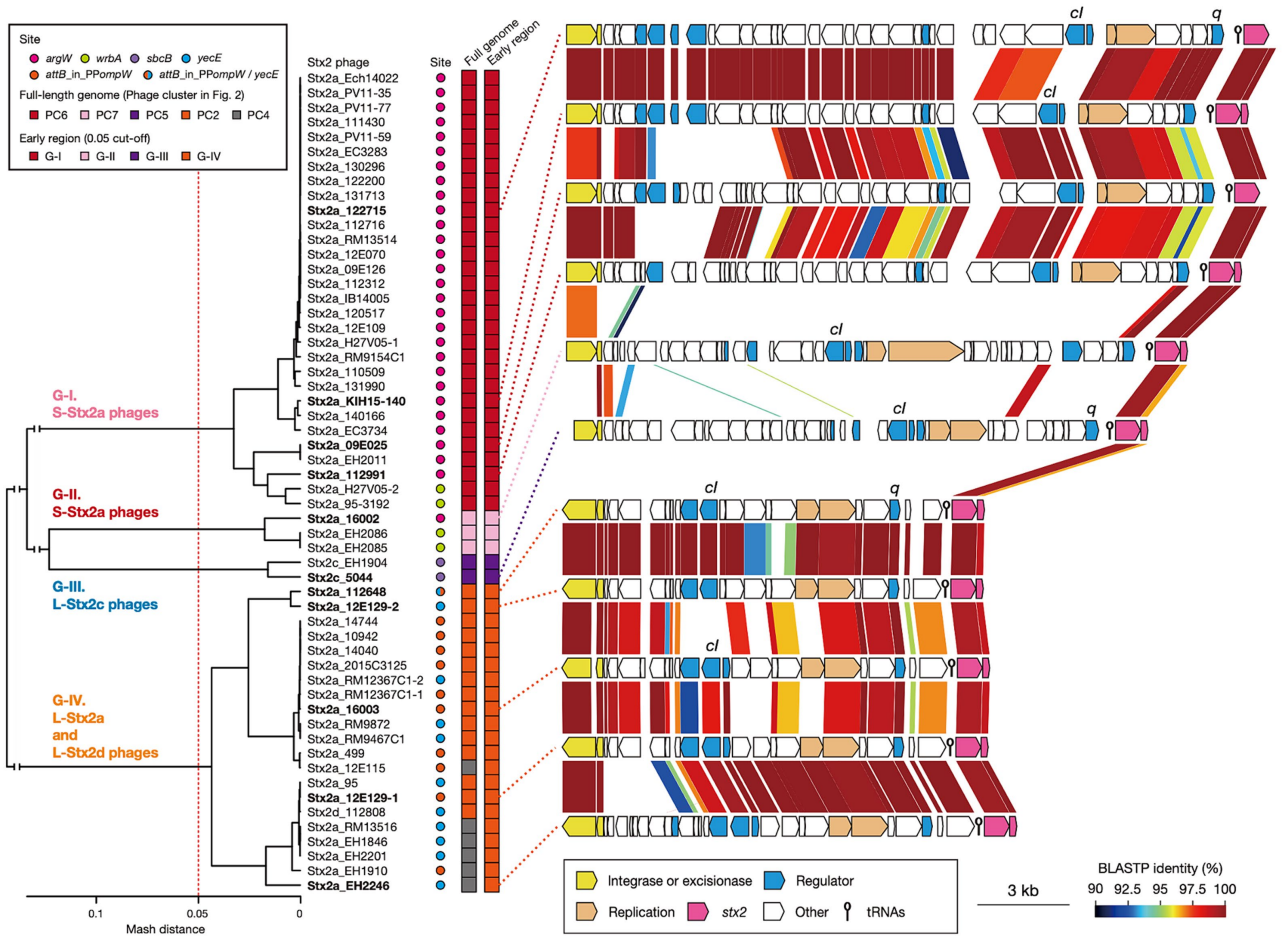
Comparisons of early regions between Stx2 phage genomes. A dendrogram constructed based on pairwise Mash distances of early regions is shown in the left panel. Stx2 phages were divided into four groups (threshold: 0.05). Phage clusters, which were defined based on the sequence similarity of full-length genomes (shown in Figure 2), and integration sites are also indicated. The genetic structures of the early regions of representative Stx2 phages (indicated in bold) are drawn to scale in the right panel. Amino acid sequence homologies are shown by shading with a heatmap.

Stx2 production levels were clearly different between the strains harboring S- and L-phages. However, as mentioned before, Stx2 production is tightly coupled with phage induction, which is achieved by the expression of early genes and the activation of late gene promoters (Wagner et al., 2001; Tyler et al., 2004). Therefore, to examine the relationship between the variation in Stx2 phages and the Stx2 production levels in host strains in more detail, we performed an additional clustering analysis of the Stx2 phages (*n* = 56; the Stx2 production levels of their host strains were determined) based on the pairwise Mash distances of their early regions (Figure 4). These phages were classified into four groups (referred to as G-I, G-II, G-III, and G-IV) with a threshold of 0.05, the same threshold that was used for the analysis of full-length phage genomes. This grouping correlated well with that based on full-length phage genomes; G-I contained all S-Stx2a phages in PC6, G-II contained all S-Stx2a phages in PC7, and G-III contained both of the two L-Stx2c phages in PC5. However, all L-Stx2a phages and one L-Stx2d phage in PC2 and PC4 were grouped together into G-IV, indicating that they contained similar early regions, which were distinct from those in the S-Stx2a and L-Stx2c phages. The sequence of Q anti-terminator, which is involved in the coupling of phage induction and Stx2 production (Tyler et al., 2004; Pacheco and Sperandio, 2012), also differed between S- and L-Stx2 phages (Supplementary Figure S4). Although the Q proteins of all S-Stx2a phages (G-I and GII groups) were similar to those of Sp5 and 933 W, the S-Stx2a phages of O157:H7 strains EDL933 and Sakai, the Q proteins of L-Stx2c phages (G-III) and those of the L-Stx2a and L-Stx2d phages (G-IV group) formed distinctive clusters except for a unique Q protein of an L-Stx2a phage in the G-IV group.

Consistent with the result of the Mash distance-based clustering analysis, the early regions exhibited distinct genetic organizations between four groups and overall sequence similarities within each group (Figure 4). However, in the G-I and G-IV groups, several genes including the *cI* gene exhibited notable within-group variations. Therefore, although the Stx2 phages of O145:H28 strains were classified into S- and L-phages, both were further divided into several groups based on the sequences of the early genes, which may have some impact on the Stx2 production levels in host strains. Similar findings related to the association of increased Stx2 production with the carriage of Stx2a phages with specific types of early regions have been reported for the S-Stx2a phages of O157:H7 STEC (Ogura et al., 2015; Miyata et al., 2023). Notably, of the L-Stx2a phages that were found in the two strains (112648 and 12E129) that carried two L-Stx2a phages and showed higher Stx2 production levels than other L-Stx2 phage-containing strains, the duplicated L-Stx2a phage in strain 112648 and one of the L-Stx2a phages in strain 12E129 formed a distinct subgroup in G-IV (Figure 4). Although the effect of the copy number of *stx* and the difference in host genetic background (ST32F and ST32H) should be considered, there is a possibility that the acquisition of these phages contributes to the increased Stx2 production level in these strains.

## Conclusion

Our systematic analysis of the full set of Stx phages in 71 STEC strains covering the entire O145:H28 lineage revealed marked genomic diversity and dynamism of Stx phages. While all Stx1 phages were long-tailed phages, Stx2 phages are divided into short-and long-tailed phages (referred to as S- and L-Stx2 phages, respectively, in this article) and the strains carrying S-Stx2 phages produced significantly more Stx2 than those carrying L-Stx2 phages. However, by detailed sequence comparison in the early region, the Stx2 phages were further classified into the four groups; G-I and G-II, each comprising S-Stx2a phages, and G-III and G-IV, each comprising L-Stx2 phages. Thus, although the variations in the early regions between S-Stx2a and L-Stx2 phages appeared to be linked to a striking difference in Stx2 production levels in the host strains and distinguishing S-Stx2a and L-Stx2a phages is important for the surveillance of STEC, the impact of the variation in the early regions within each group and the carriage of two Stx2 phages on the levels of Stx2 production by host strains should be further analyzed in future.

## Data availability statement

The datasets presented in this study can be found in online repositories. The names of the repository/repositories and accession number(s) can be found in the article/Supplementary material.

## Author contributions

KN: Conceptualization, Formal analysis, Funding acquisition, Methodology, Visualization, Writing – original draft, Writing – review & editing. IT: Formal analysis, Software, Writing – review & editing. YG: Formal analysis, Writing – review & editing. JI: Resources, Writing – review & editing. KK: Resources, Writing – review & editing. YI: Resources, Writing – review & editing. TK: Resources, Writing – review & editing. YT: Resources, Writing – review & editing. RN: Resources, Writing – review & editing. KI: Resources, Writing – review & editing. YM: Resources, Writing – review & editing. SI: Resources, Writing – review & editing. TH: Conceptualization, Funding acquisition, Project administration, Supervision, Writing – original draft, Writing – review & editing.

## Footnotes

^1^ http://tree.bio.ed.ac.uk/software/figtree/
