# Supplementary material for: Diversity of Shiga toxin transducing phages in Escherichia coli O145:H28 and the different Shiga toxin 2 production levels associated with short- or long-tailed phages
Source: Front Microbiol. 2024 Aug 6;15:1453887. doi: 10.3389/fmicb.2024.1453887 (PMC11333237; doi:10.3389/fmicb.2024.1453887)
Supplement: Supplementary file 1 [file Data_Sheet_1.PDF]

A

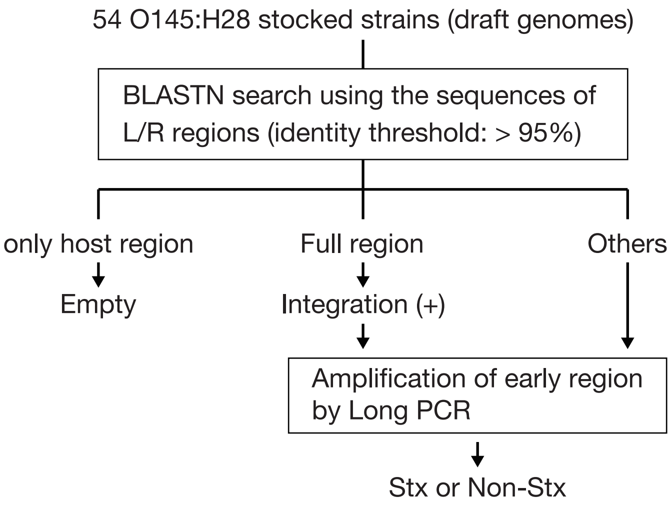

Region	strain	positions
*wrbA*_L	95-3192	2,461,497 - 2,461,623
*wrbA*_R	95-3192	2,397,055 - 2,397,181
*argW*_L	122715	3,045,002 - 3,045,145
*argW*_R	122715	2,982,390 - 2,982,533
*sbcB*_L	RM13514	2,610,545 - 2,610,676
*sbcB*_R	RM13514	2,565,170 - 2,565,301

B

Type	Site	Strain
I	*wrbA*	10 strains*
II	*wrbA*	EH2085, EH2086 and H27V05
III	*wrbA*	EH2201, EC3734
IV	*argW*	26 strains†
V	*sbcB*	121427
VI	*sbcB*	EH1904 and 5044
VIIa	*sbcB*	699, PV11-35, EH2201, 09E025 and IB14005
VIIb	*sbcB*	H27V05, KIH15-140, EC3734, 140166, Ech14022 and 12E129

Strategy for amplification of prophage region

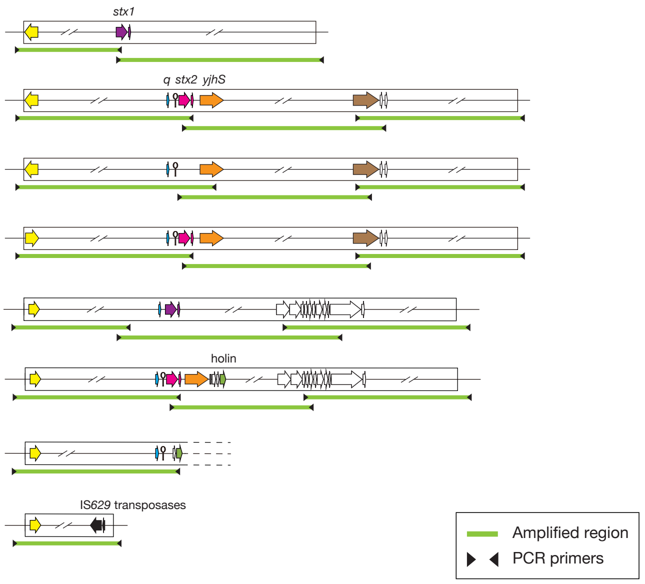

* Stx1a phages of strains 9793, 111698, 131719, 8100, 1649, 1380, 120412, 112808, EH2197 and 12188

† Stx2a phages of strains EC3283, PV11-77, 111430, PV11-59, 130296, 131990, 122200, 131713, 110509, 112312, 112716, 120517, 09E126, 12E070, PV11-35, 12E109, 09E025, 16002, 112991, EH2011, IB14005, H27V05, KIH15-140, EC3734, 140166 and Ech14022

Supplementary Figure S1

Procedures to determine the integration sites and genome sequences of Stx phages in the O145:H28 strains, for which only draft genomes were available.

(A) Determination of integration sites by BLASTN search. Draft genomes of O145:H28 strains (n=54) were searched by BLASTN using the six query sequences; the *attL*-flanking and *attR*-flanking sequences from each of the prophage-integrated *wrbA* locus in strain 95-3192 (*wrbA*_L/R), the prophage-integrated *argW* locus in strain 122715 (*argW*_L/R), and the prophage-integrated *sbcB* locus in strain RM13514 (*sbcB*_L/R). Each query sequence was composed of the *att* sequence (7 bp for the prophage at *wrbA*, 24 bp for the prophage at *argW* and 12 bp for the prophage at *sbcB*) and the host chromosome and prophage sequences (60 bp each) that flanked the *att* sequence. Phage integration at each locus was considered positive when both *attL*- and *attR*-flanking sequences were detected (identity threshold: >95%). Integration sites of Stx phages in all but one genomes were determined by this procedure. In strain IB14005, we detected the *argW*_L sequence but not the *argW*_R sequence. Therefore, the *argW* locus of this strain was defined as 'Others' and subjected to long PCR analysis.

(B) Strategies for the long PCR and sequence determination of Stx phage genomes are shown. Stx prophage regions in each strain were divided into two or three segments and amplified by long PCRs as indicated and PCR products obtained from each strain were subjected to Illumina sequencing to determine the sequence of the entire prophage region. The complete sequences of two non-Stx phages integrated into *wrbA* , the sequences of the early regions of five non-Stx phages integrated into *sbcB*, and the full-length sequences of six degraded non-Stx phages integrated into *sbcB* were also determined by Type III, Type VIIa, and Type VIIb strategies, respectively.

A

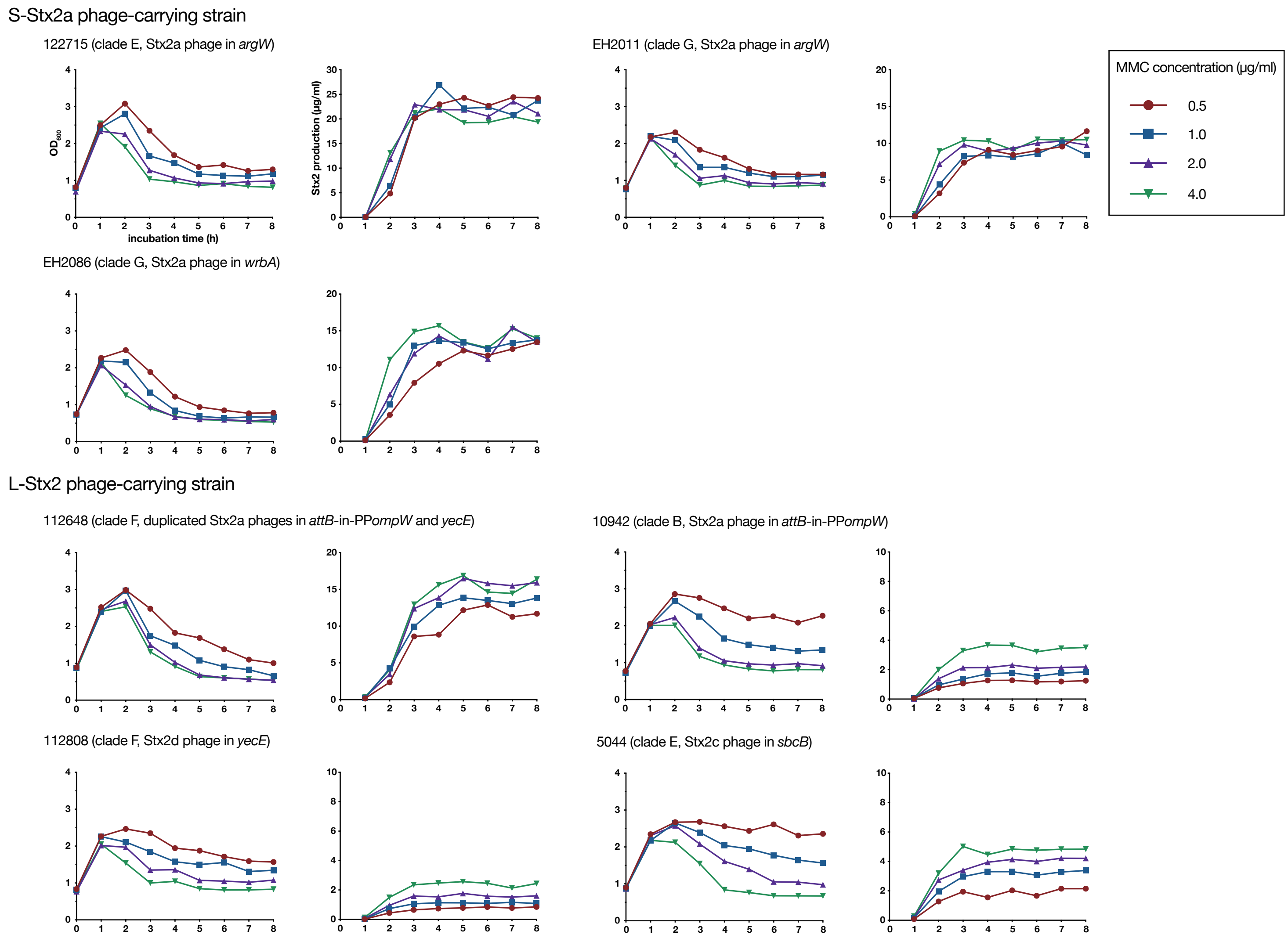

B

	O145:H28 strain							STEC strain (Serotype)						
	122715	EH2011	EH2086	112648	10942	112808	5044	Sakai (O157:H7)	11368 (O26:H11)	11128 (O111:H8)	12009 (O103:H2)	51104 (O121:H19)	K-12	ATCC 25922
MIC (µg/ml)	4	4	2	2	4	4	2	2	2	2	4	2	4	2

Supplementary Figure S2

Optimization of the Stx2 production assay.

(A) Lysis curves and Stx2 concentrations of seven O145:H28 strains (three strains carrying S-Stx2a phages and four strains carrying L-Stx2 phages). In each strain, the MMC-induced lysis curve (left) and the Stx2 concentrations in cell lysates obtained at each time point (right) are shown. Bacterial cells were inoculated into 40 ml of LB at a cell concentration of 0.1 OD_{600} and grown to mid-log phage at 37 °C with shaking. MMC was added to the culture at a final concentration of 0.5, 1.0, 2.0, or 4.0 µg/ml. After the addition of MMC, the OD_{600} of each culture was measured every hour for 8 h, and 100 µl of the culture was collected at each time point to prepare cell lysates. The Stx2 concentration in each lysate was determined by sandwich ELISA (n=1). In most cases, the maximum cell lysis and the highest Stx2 concentration were observed at 6 h. (B) Minimum inhibitory concentrations (MICs) of various *E. coli* strains against MMC. The MMC susceptibilities of seven O145:H28 strains, five STEC strains belonging to other major STEC serotypes, and strains K-12 and ATCC25922 were determined according to the standard protocol outlined in the CLSI (Clinical and Laboratory Standards Institute) guidelines (M100 Performance Standards for Antimicrobial Susceptibility Testing, 28th Edition, CLSI 2018). The MICs of all tested strains were 2.0 µg/ml or 4.0 µg/ml. Based on these results, we decided to use the concentration of 1 µg/ml for MMC, which was lower than MIC and induced higher levels of Stx2 production than 0.5 µg/ml of MMC in most strains.

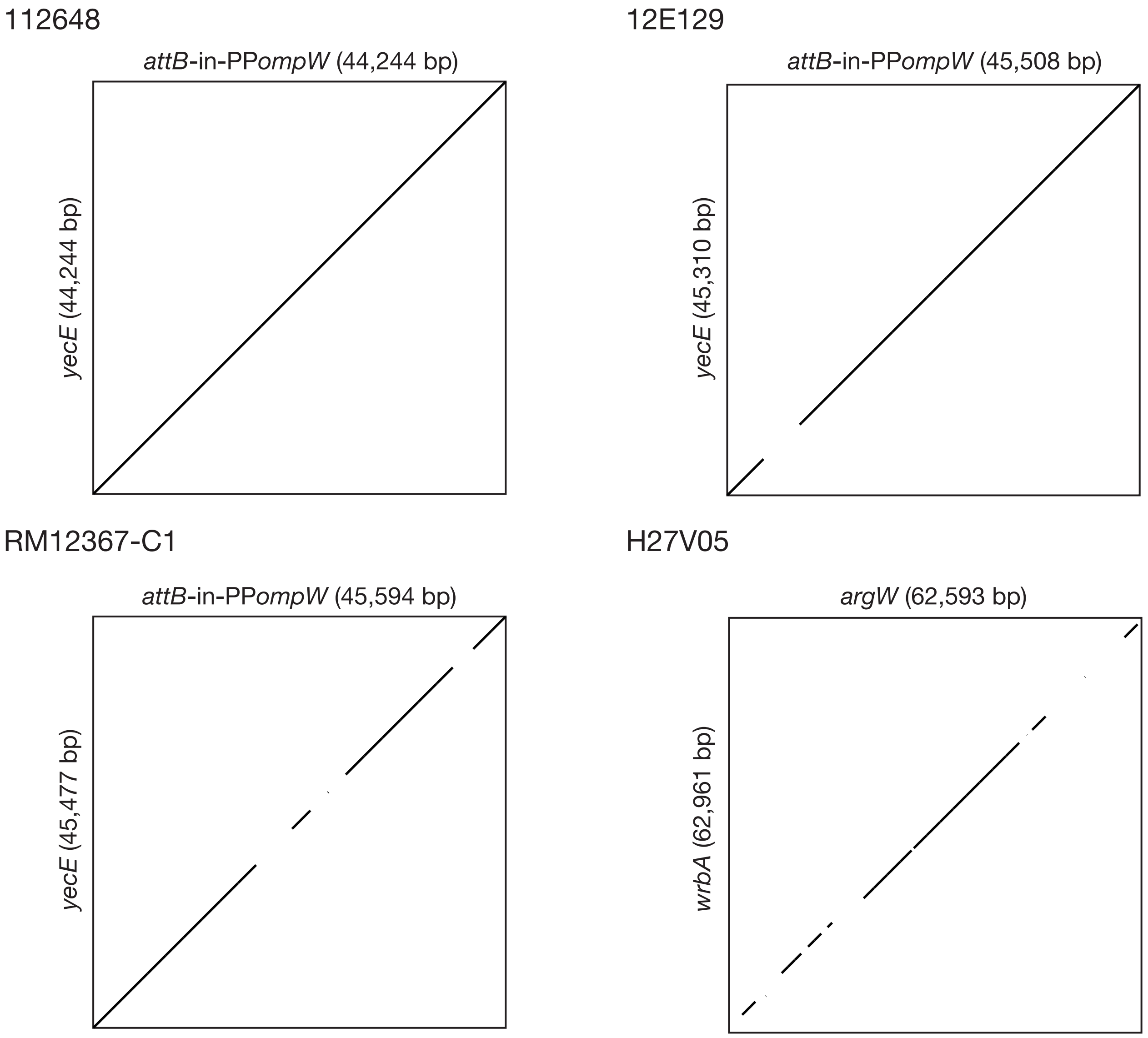

Supplementary Figure S3

Sequence similarities of the two Stx2a phage genomes found in each of the four O145:H28 strains.

Dot plot presentation of sequence similarity (window size of 2 kb; >99% sequence identity) between two Stx2a phages in each of strains 112648, 12E129, RM12367-C1, and H27V05 are shown. The integration site and genome size of each phage are indicated in X- and Y-axes. Note that the two Stx2a phages in strain 112648 are completely identical.

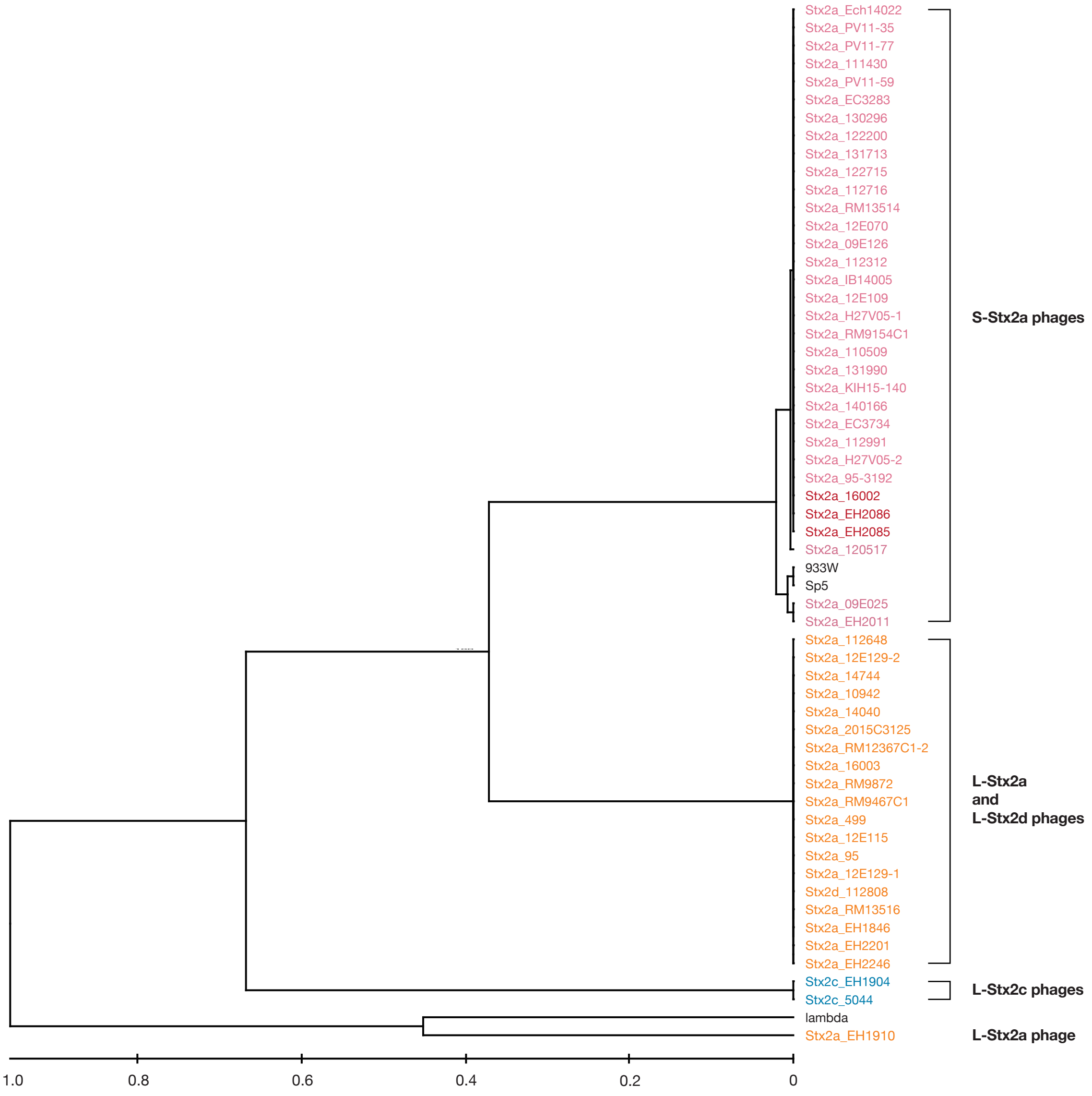

Supplementary Figure S4

Variation in the sequences of Q antiterminator proteins encoded by the Stx2 phages of O145:H28 strains.

A UPGMA tree was generated based on the alignment of amino acid sequences of Q proteins. The Q proteins of phages lambda (No. NP_040642.1), Sp5 (No. BAA94139.1), and 933W (No. NP_049499.1) were included as references. The Stx2 phages of O145:H28 strains were colored according to the groups shown in Figure 4 (G-I, pink; G-II, red; G-III, blue; G-IV, orange). The Q protein encoded by the L-Stx2a phage of strain RM12367C1-1, which contained a frameshift mutation, was excluded from this analysis.
